# Differential Allocation of Constitutive and Induced Chemical Defenses in Pine Tree Juveniles: A Test of the Optimal Defense Theory

**DOI:** 10.1371/journal.pone.0034006

**Published:** 2012-03-28

**Authors:** Xoaquín Moreira, Rafael Zas, Luis Sampedro

**Affiliations:** 1 Misión Biológica de Galicia, Consejo Superior de Investigaciones Científicas, Pontevedra, Galicia, Spain; 2 Centro de Investigación Forestal de Lourizán - Unidad Asociada a la Misión Biológica de Galicia, Xunta de Galicia, Pontevedra, Galicia, Spain; Max Planck Institute for Chemical Ecology, Germany

## Abstract

Optimal defense theory (ODT) predicts that the within-plant quantitative allocation of defenses is not random, but driven by the potential relative contribution of particular plant tissues to overall fitness. These predictions have been poorly tested on long-lived woody plants. We explored the allocation of constitutive and methyl-jasmonate (MJ) inducible chemical defenses in six half-sib families of *Pinus radiata* juveniles. Specifically, we studied the quantitative allocation of resin and polyphenolics (the two major secondary chemicals in pine trees) to tissues with contrasting fitness value (stem phloem, stem xylem and needles) across three parts of the plants (basal, middle and apical upper part), using nitrogen concentration as a proxy of tissue value. Concentration of nitrogen in the phloem, xylem and needles was found to be greater higher up the plant. As predicted by the ODT, the same pattern was found for the concentration of non-volatile resin in the stem. However, in leaf tissues the concentrations of both resin and total phenolics were greater towards the base of the plant. Two weeks after MJ application, the concentrations of nitrogen in the phloem, resin in the stem and total phenolics in the needles increased by roughly 25% compared with the control plants, inducibility was similar across all plant parts, and families differed in the inducibility of resin compounds in the stem. In contrast, no significant changes were observed either for phenolics in the stems, or for resin in the needles after MJ application. Concentration of resin in the phloem was double that in the xylem and MJ-inducible, with inducibility being greater towards the base of the stem. In contrast, resin in the xylem was not MJ-inducible and increased in concentration higher up the plant. The pattern of inducibility by MJ-signaling in juvenile *P. radiata* is tissue, chemical-defense and plant-part specific, and is genetically variable.

## Introduction

Distribution of anti-herbivore defenses within a plant is expected not to be homogeneous. With chemical defenses in particular, different compounds could be present at different concentrations in different plant parts and tissues. Optimal Defense Theory (ODT) was originally developed to explain the distribution of chemical and morphological defenses within a plant [1], [2]. The basic assumption of ODT is that defenses are costly to produce and thus could trade-off with other plant functions, such as growth and/or reproduction [3]. Because defenses are costly, ODT predicts that plants would have the highest levels of chemical defenses in those tissues or organs that have the highest value in terms of fitness and/or those tissues that are more frequently attacked by herbivores [4]. For example, young growing tissues (e.g. upper meristematic and leaf tissues) have been suggested to be more valuable to plant fitness than old tissues (e.g. tissues in lower parts of the plant) and often they have been shown to have greater concentrations of chemical defenses [5]–[8]. Similarly, reproductive tissues such as flowers and fruits, which are not so easy to replace as vegetative tissues, frequently have greater chemical defenses against herbivory [9]–[11]. Thus, a basic prediction of ODT is that defensive function should be distributed in a heterogeneous pattern across plant tissues and plant parts differing in value, cost or risk of attack. This prediction has been tested and frequently confirmed in annual and herbaceous plants [6], [8], [12]–[14], in seaweeds [15], [16] and even in lichens [17], but few studies have examined it's veracity for long-lived woody plants [18], with life history determinants greatly different to those of annual and herbaceous plants.

Most of the studies evaluating the differential allocation of chemical defenses within plant tissues have either considered only the preformed (constitutive) defenses, or have not differentiated between constitutive and herbivore-inducible defensive mechanisms. As most plant defenses are plastic traits, inducible after attack or in the presence of herbivore cues, inducibility of chemical defenses could also differ depending on plant part and tissue. Induced defenses are cost saving strategies, in which the costs of the production of defenses are materialized only when needed, i.e. after an attack [19], [20]. It could thus be expected that in order to invest resources efficiently, the inducibility of chemical defenses might vary across plant parts or tissues, depending on the fitness value and the costs of production of every given tissue, and also on the predictability and risk of attack and expected herbivore loadings [10], [16], [21], [22].

In this paper, we hypothesize that, according to the predictions of the ODT, allocation patterns of constitutive and induced quantitative chemical defenses in pine trees would differ among plant parts and tissues. A pattern of differential allocation would be particularly beneficial during the early stages of the pine tree life, when resources are scarce and herbivory could have extreme consequences in terms of survival, and thus of fitness [23]. For this purpose we performed a greenhouse experiment with two-year-old juveniles of *Pinus radiata* D. Don. belonging to six half-sib families. We mimicked herbivore-induced responses using methyl jasmonate (MJ), a phytohormone that elicits defensive responses similar to those induced by herbivore wounding in conifer trees (e.g. [24]–[26]). We examined the strategy of constitutive and induced allocation of the two major quantitative chemical defenses of conifers, oleoresin and polyphenolics [27], to three tissues with contrasting fitness value (stem phloem, stem xylem and needles) across three parts of the plants (basal, middle and apical upper part). We used N concentration as a proxy of value of the same tissues and plant parts, according to Traw & Feeny [7] and McCall & Fordyce [8]. We hypothesize that young growing apical tissues should be the better defended targets during the juvenile stages of a fast-growing sun-demanding pine tree. In addition, a differential effort would be expected between xylem and phloem, as the latter is the first protective barrier against stem borers and the main target for wounding insects.

## Materials and Methods

### Ethics Statement

The research did not involve measurements on humans or animals. No specific permissions were required for our field work. The plant material used for this study was only sampled at a very limited scale and therefore had negligible effects on broader ecosystem functioning. The location is not privately-owned or protected in any way. The field studies did not involve endangered or protected species.

### Experimental design

We conducted a greenhouse experiment following a randomized split-plot design replicated in six blocks, with MJ-induction of defensive responses (two levels: MJ-treatment and control) as the whole factor, and genetic entries (six open-pollinated half-sib families, known mother trees) as the split factor, in order to account for possible genetic variation and interactions. In total, there were 72 pine juveniles, corresponding to 6 blocks ×2 MJ treatments ×6 genetic entries.

### Plant material, greenhouse conditions and MJ-induction

The plant material consisted of open-pollinated families from 6 maternal plus trees selected for superior growth and form in mature plantations of *P. radiata* in Galicia (NW Spain). In April 2006, seeds were individually sown in 2 L pots filled with peat and perlite (1∶1 v∶v), fertilized with 12 g of a slow release fertilizer (Multicote® N∶P∶K 15∶15∶15), and grown in an isolated glass greenhouse with controlled light (minimum 12 h per day), and temperature (10°C night, 25°C day) and daily watering.

In July 2008, approximately two years after sowing (plant height 61.35±1.89 cm), half of the plants were treated with a solution of 80 mM MJ (ref #39270-7, Sigma-Aldrich, St. Louis, MO, US) in deionised water with ethanol 2.5% (v∶v). The remaining plants were treated only with the carrier solution, acting as a control. Each plant received 5.2±0.8 ml of solution applied to the foliage with a handheld sprayer to runoff. Dose and concentration of MJ solution were determined based on plant size and previous experience with pine juveniles [28]. To avoid cross-contamination, treatments were applied in separate greenhouse chambers and plants remained in those separate spaces for 48 h to allow drying.

### Sampling and measurements

On August 2008, two weeks after MJ application, pines were harvested and transported to the lab in ice coolers. Then, the whole stem was immediately cut into 3 parts of the same length (basal, middle and apical upper part). Length and diameter of each part were measured, and a fresh 10 cm-long piece of the stem and a subsample of needles (approximately 4 g) of each plant across the three above-described parts were sampled, weighed, immediately frozen and preserved at −30°C for analysis of resin. A fresh 2 cm-long piece of the stem and a subsample of needles (approximately 2 g) of the basal, middle and apical upper parts were also weighed, oven-dried (45°C to constant weight) and then manually ground in a mortar with liquid nitrogen for analyses of total phenolic compounds. In a subsample of two randomly selected pine families in four blocks (N = 16 plants), an additional fresh 5 cm-long piece of the stem across the three above-described parts was sampled, weighed, carefully separated into phloem and xylem with a scalpel, immediately frozen and preserved at −30°C for analysis of resin in the phloem and xylem. In a subsample of two randomly selected pine families in four blocks (N = 16 plants), an additional fresh 2 cm-long piece of the stem (separated into phloem and xylem) and a subsample of needles (approximately 1 g.) across the three above-described parts were sampled, oven dried for 72 h at 65°C to constant weight, finely ground and stored for the analysis of N content. Due to sample loss, N concentration in the xylem was analyzed only in one pine family in four blocks (sample size N = 8 plants).

### Chemical analysis

Total phenolics were extracted and analyzed as described in Sampedro et al. [29]. Briefly, phenolics were extracted from 300 mg of plant tissue with aqueous methanol (1∶1 vol∶vol) in an ultrasonic bath for 15 min, followed by centrifugation and subsequent dilution of the methanolic extract. Total phenolic content was determined colorimetrically by the Folin-Ciocalteu assay in a Biorad 650 microplate reader (Bio-Rad Laboratories, Philadelphia, PA, USA) at 740 nm, quantified with a standard curve of tannic acid and expressed as mg tannic acid equivalents g^−1^ dry mass of plant tissue.

Concentration of resin was estimated gravimetrically as described in Sampedro et al. [29], and expressed as mg of non-volatile resin g^−1^ stem dried weight (d.w.). Briefly, plant material was transferred into preweighed tubes, resin was extracted with 3 ml of hexane (15 min at 20°C in an ultrasonic bath and then for 24 h at room temperature), the extract was filtered (Whatman GF/D, Whatman Int. Ltd, Maidstone, Kent, UK) into preweighed tubes, and the whole extraction step repeated again in new tubes. The solvent in the tubes was evaporated to dryness and the mass of the non-volatile resin residue was determined at the nearest 0.0001 g. Estimates of resin content using this simple procedure were found to correlate well with diterpene content (r = 0.9214; P = 0.0001; N = 20) as determined by gas chromatography [29].

Total N was determined with a CN-2000 macro elemental analyzer (LECO Corporation, St. Joseph, MI, USA) at the central facilities of Universidade de Vigo, Spain (http://webs.uvigo.es/cactiweb/).

### Statistical analyses

The effects of design factors were analyzed with a repeated measures mixed model with restricted maximum likelihood using the PROC-MIXED procedure of the SAS System. Methyl jasmonate induction (MJ), family (G), plant part (P), block (B), and the interactions between MJ, G and P were considered fixed factors. The B×MJ interaction was considered a random factor in order to analyze the main factor MJ in the split-plot design with the appropriate error term [30]. Plant part and the corresponding interactions were considered as within-subject factors. Equality of residual variance across treatments was tested in all cases, but significant deviations were not found. When main effects were significant differences between MJ vs control treatments and among plant parts were tested for significance using the LSMEAN statement. Data are shown as means ± standard error of the mean (s.e.m.).

## Results

**Figure 1:**
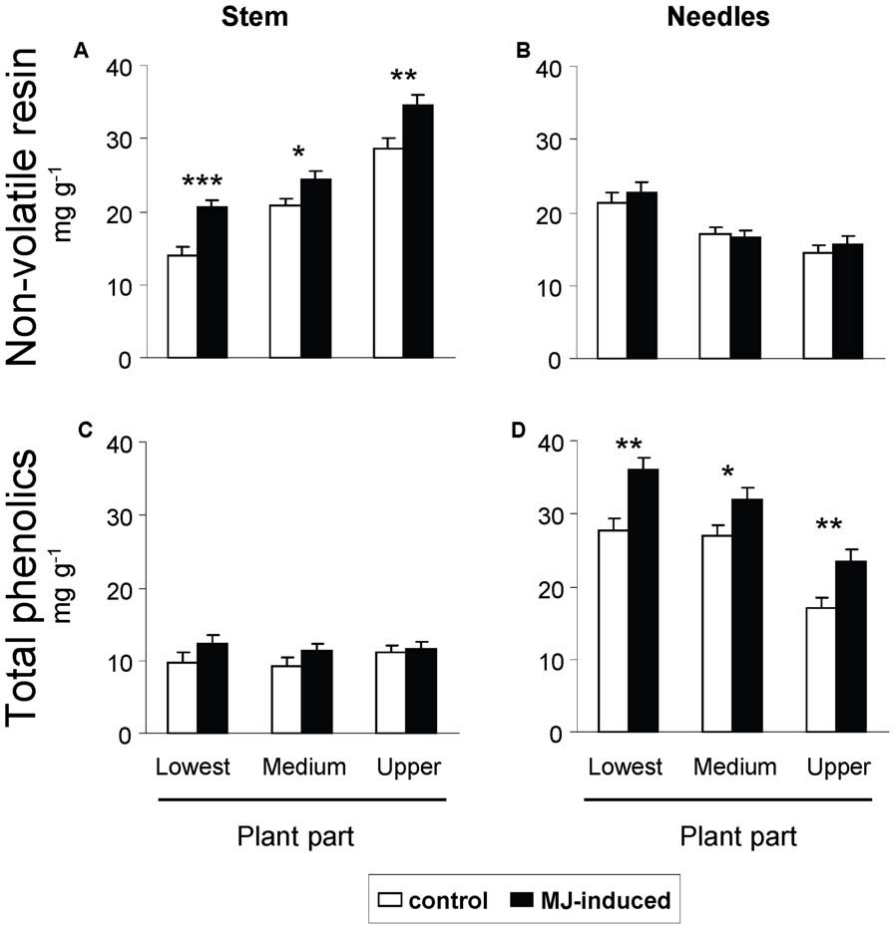
Allocation of constitutive and induced chemical defenses across different pine parts. Concentration of non-volatile resin in (a) the stem and (b) the needles, and total phenolics in (c) the stem and (d) the needles across three parts of the plants (basal, middle and apical upper part) in control (constitutive, white bars) and methyl-jasmonate (MJ) induced (black bars) *P. radiata* juveniles. Plants were destructively sampled 15 days after application of MJ. Bars are means ± s.e.m. (N = 36). Asterisks indicate significant differences within each plant part due to MJ-induction at *P*<0.05 (*), *P*<0.01 (**) and *P*<0.001 (***).

Allocation of secondary chemicals significantly differed among the plant parts (Table 1): resin concentration in the stem tissues was greater towards the top of the plant (Fig. 1a), whereas in leaf tissues resin and phenolics content were both greater towards the base of the plant (Fig. 1b, 1d). Stem phenolic concentrations were low relative to needles and did not show significant changes across different parts of the stem (Table 1, Fig. 1c). Application of MJ significantly affected the concentrations of resin in the stem and total phenolics in the needles (Table 1), but not those of resin in the needles and total phenolics in the stems (Table 1; Fig. 1b, 1c). Specifically, MJ-induced concentrations of stem resin and needle total phenolics were 25% and 30% greater, respectively, than those in control plants (Fig. 1a, 1d). Interestingly, inducibility of total phenolics and resin compounds did not differ among plant parts (non-significant MJ×part interaction, Table 1).

**Table 1:** Allocation of constitutive and induced chemical defenses across different pine parts.

		Stem				Needles			
		resin		phenolics		resin		phenolics	
	DF^1^ (n,d)	F	*P*	F	*P*	F	*P*	F	*P*
MJ-induction	1, 5	41.32	**0.001**	2.21	0.197	0.27	0.626	22.10	**0.005**
Genotype (G)	5, 50	1.29	0.283	0.45	0.811	0.44	0.811	1.34	0.262
MJ×G	5, 50	2.41	**0.049**	0.53	0.752	1.68	0.156	1.85	0.120
Plant part (P)	2, 115	129.22	**<0.001**	1.72	0.184	26.93	**<0.001**	42.29	**<0.001**
MJ×P	2, 115	1.50	0.227	0.81	0.449	0.92	0.402	0.87	0.420
G×P	10, 115	1.44	0.171	0.83	0.600	1.21	0.291	1.09	0.373
MJ×G×P	10, 115	2.96	**0.002**	0.92	0.517	0.59	0.822	0.95	0.492

1 DF = degrees of freedom (numerator, denominator).

Summary of the repeated measures mixed model for constitutive and methyl-jasmonate (MJ) induced allocation of chemical defenses (non-volatile resin and total phenolics) to two tissues (stem and needles) across three parts of the plants (basal, middle and apical upper part) in six *P. radiata* open-pollinated families. Significant *P* values (*P*<0.05) are typed in bold.

In our experiment we used a reduced sample of families, and we did not find significant differences between families in the overall concentration of defenses across parts and tissues. However, significant differences between families were found in terms of the inducibility of resin in the stem (although not of total phenolics) (MJ×G; *P*<0.05; Table 1), that was consistent with the significant three-way interaction (MJ×G×P, Table 1), indicating differences among families in the inducibility of resin across the different parts of the stem. We observed some differences between families in terms of inducibility of resin in the upper part of the stem, and in the case of one interacting family in the basal part, but no corresponding differences in the middle part of the stem (data not shown).

**Figure 2:**
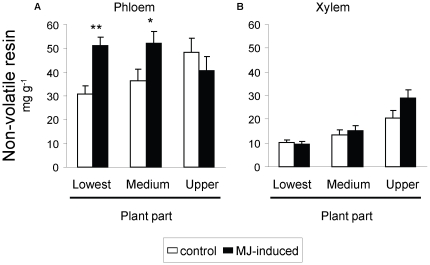
Allocation of constitutive and induced non-volatile resin across different pine parts. Non-volatile resin concentration in (a) the phloem and (b) the xylem across three parts of the plants (basal, middle and apical upper part) in control (constitutive, white bars) and methyl jasmonate (MJ) induced (black bars) *P. radiata* juveniles. Plants were destructively sampled 15 days after application of MJ. Bars are means ± s.e.m. (N = 8). Asterisks indicate significant differences within each plant part due to MJ-induction at *P*<0.05 (*) and *P*<0.01 (**).

We observed that concentration of resin in the xylem was markedly greater towards the top of the plant (Table 2; Fig. 2b), while on the other hand resin concentration in the phloem, which was much greater than that in the xylem, did not show significant changes across different plant parts (Table 2; Fig. 2a). Application of MJ did not significantly affect overall concentration of resin in the xylem (Table 2). However MJ increased significantly the phloem resin concentration in the basal and middle parts of the stem, but not in the upper part (MJ×part significant interaction; Table 2; Fig. 2a).

**Table 2:** Allocation of constitutive and induced non-volatile resin across different pine parts.

		Phloem resin		Xylem resin	
	DF^1^ (n,d)	F	*P*	F	*P*
MJ-induction	1,3	5.60	0.099	2.63	0.203
Genotype (G)	1,6	2.92	0.139	0.10	0.763
MJ×G	1,6	0.13	0.732	0.71	0.431
Plant part (P)	2,24	0.38	0.688	20.18	**<0.001**
MJ×P	2,24	5.12	**0.014**	2.08	0.147
G×P	2,24	1.84	0.181	0.49	0.616
MJ×G×P	2,24	1.13	0.341	0.04	0.960

1 DF = degrees of freedom (numerator, denominator).

Summary of the repeated measures mixed model for constitutive and methyl-jasmonate (MJ) induced allocation of non-volatile resin to two tissues (phloem and xylem) across three parts of the plants (basal, middle and apical upper part) in two *P. radiata* open-pollinated families. Significant *P* values (*P*<0.05) are typed in bold.

**Figure 3:**
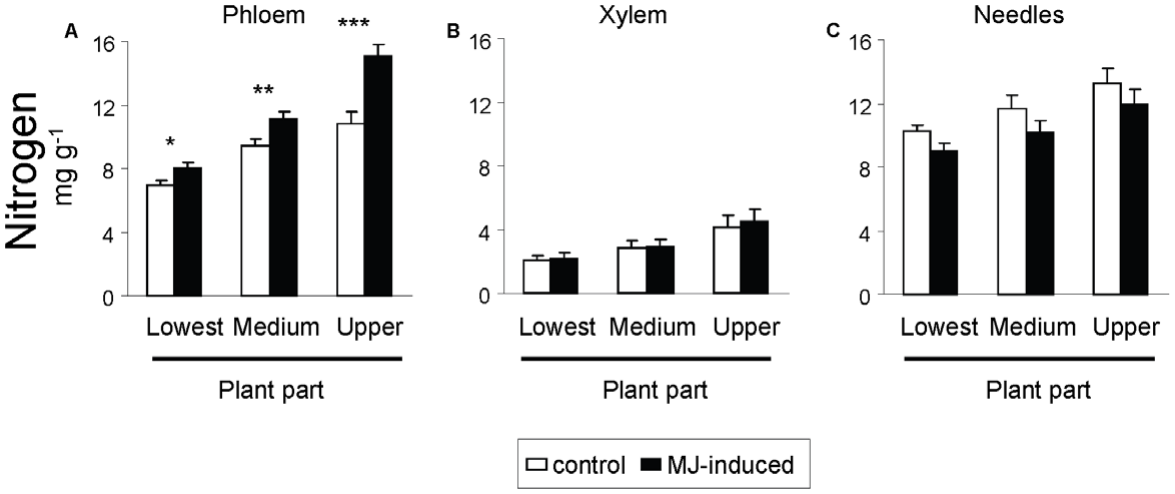
Allocation of constitutive and induced concentration of nitrogen across different pine parts. Nitrogen concentration in (a) the phloem, (b) the xylem and (c) the needles across three parts of the plants (basal, middle and apical upper part) in control (constitutive, white bars) and methyl jasmonate (MJ) induced (black bars) *P. radiata* juveniles. Plants were destructively sampled 15 days after application of MJ. Bars are means ± s.e.m. (N = 12 for phloem and needle tissues and N = 6 for xylem tissue). Asterisks indicate significant differences within each plant part due to MJ-induction at *P*<0.05 (*), *P*<0.01 (**) and *P*<0.001 (***).

Finally, we also found that the concentration of N in the phloem, xylem and needles increased higher up the plant (Table 3; Fig. 3a, 3b, 3c). Application of MJ significantly affected N concentration in the phloem, prompting a 25% increase with respect to control plants two weeks after treatment application (Table 3; Fig. 3a), This increase in overall concentration was accompanied by changes in their allocation pattern, as revealed by the significant MJ×part interaction (Table 3). Specifically, the rise of N concentration was significantly greater in the apical upper part (Fig. 3a). MJ application did not affect N concentration of needles and xylem tissues (Table 3; Fig. 3b, 3c).

**Table 3:** Allocation of constitutive and induced concentration of nitrogen across different pine parts.

		Phloem nitrogen		Needle nitrogen		Xylem nitrogen		
	DF^1^ (n,d)	F	*P*	F	*P*	DF^1^ (n,d)	F	*P*
MJ-induction	1,3	16.83	**0.031**	2.83	0.191	1,3	0.30	0.624
Genotype (G)	1,6	6.48	**0.044**	1.11	0.333			
MJ×G	1,6	1.19	0.318	0.17	0.692			
Plant part (P)	2,24	66.04	**<0.001**	11.18	**<0.001**	2,12	25.48	**<0.001**
MJ×P	2,24	6.57	**0.005**	0.07	0.936	2,12	0.17	0.849
G×P	2,24	0.64	0.536	1.30	0.290			
MJ×G×P	2,24	2.34	0.117	0.53	0.596			

1 DF = degrees of freedom (numerator, denominator).

Summary of the repeated measures mixed model for constitutive and methyl-jasmonate (MJ) induced nitrogen concentration in three tissues (phloem, xylem and needles) across three parts the plants (basal, middle and apical upper part). Sample size was N = 12 except for xylem, that was N = 6 due to sample loss, resulting in only one genetic entry analyzed. Significant *P* values (*P*<0.05) are typed in bold.

## Discussion

Our results show the existence of a marked gradient of quantitative allocation of chemical defenses within juvenile pines, with different patterns for phenolics and resin in stems and needles between the basal, middle and upper pine parts. Specifically, the apical upper part of the stem was better defended with resin compounds than the basal and middle sections, whereas the concentration of total phenolics and resin in the needles was greater in the basal part of the plants. Our results also showed a marked gradient of N allocation within the plant tissues (phloem, xylem and needles), with increasing concentrations observed higher up the plant. Nitrogen availability is an important constraint on seedling growth of fast-growing species and there is considerable literature reporting greater N concentrations in the young and growing tissues of the uppermost plant parts, (e.g. [31]), and as such N concentration is a useful proxy for determining plant tissue value [7], [8]. Other authors have assessed tissue fitness value by removing different plant tissues and measuring the impact on subsequent plant fitness [6], [12]. However, although removal of needles without apparent defensive responses have been used elsewhere, we think that removal of plant tissues in pine trees could induce herbivory signaling and provoke defensive responses, potentially leading to dramatic changes in the plant sink-source balance, carbon allocation and growth patterns [32]–[34]. This is of particular relevance given that our aim is to study constitutive and induced defenses, and could well lead to confounding results.

The ODT postulates that the allocation to defenses within a plant will be highest in those tissues with greatest value to the plant [1], [2]. Fitting with this prediction, we found that the distribution of resin along the pine stem was greater in the upper and younger growing meristematic tissues, which were precisely those that had greater N concentration. However, concentration of chemical defenses in the needles (both resin and phenolics) was greater in the basal part of the plants. One explanation for this pattern arises from the fact that N allocation among the leaves is optimized with respect to photosynthetic production [35]. Plant resources for growth are obtained from middle and basal photosynthesizing leaves (sources) and are translocated to upper newly growing tissues (sink) [36]. Thus, one-year-old needles in the lower part of the plant may support the majority of a pine's photosynthetic capacity and in consequence need to be better defended than newly growing needles. Another possible explanation for these results could be the ontogeny delay in needles, whereby the young needles in the apical part may simply not have yet had enough time to accumulate as high levels of defensive chemicals as in lower needles. The fitness value of different plant parts change over space and time and thus defense allocation is also predicted to change during ontogeny [6], [37]–[39]. Further studies should address differential allocation of defenses in older stages of the pine tree life.

The second prediction of ODT is that the allocation to defenses within a plant will be highest in those tissues with greatest risk of attack from consumers. The juveniles of *P. radiata* support a diverse array of enemies, including root pathogens, aphids, bark beetles, weevils, borers, caterpillars, and moths [40], [41]. We lack data about the predictability and risk of attack by those insects and pathogens, and their effects on *P. radiata* survival or fitness in its reduced natural range. However, even though it is difficult to estimate the risk of attack, in other pine species it is known that the survival of young pines is seriously threatened when the lower part of the stem is subjected to herbivory by phloem chewers such as the large pine weevil *Hylobius abietis*, an occasional but problematic pest for European conifer forests. This insect causes massive mortalities due to intense wounding at the basal part of the stem, frequently producing stem girdling that cuts the sap flow and consequently leads to plant death [e.g. 42]. This idea seems to agree with the greater inducibility of phloem resin observed in the lower part of the stem of our pine trees (Fig. 2a). Considering the strong induced resin production observed soon after herbivory by this insect in other pine species [43], future research should address what defenses are effective against each of the pine's natural enemies and the allocation patterns of pine defenses in response to particular biotic challenges.

Our results also showed that the concentrations of non-volatile resin in the stem and total phenolics in the needles were responsive to MJ-induction, but conversely concentrations of phenolics in the stem and resin in the needles were unresponsive. The increases in concentration of chemical defenses are consistent with the role of MJ in signaling defensive responses that have been extensively studied in young conifer trees (e.g., [24], [26], [28], [29], [44]). For example, Sampedro et al. [29] observed that exogenous application of MJ heavily increased the concentration of resin in the stem and total phenolics in the needles of *P. pinaster* juveniles. That stem and needles should differ in their responses to MJ application also agrees with previous studies that observed no changes or only minor alterations in needle terpenoids in young conifer trees when compared with other tissues of the same plants, such as stem wood and roots [25], [45], [46]. We also observed that the increase in N concentration in the phloem was greater in the apical upper part of the plant. Changes in the allocation pattern of N over the height of the plant after real herbivory or herbivory signaling have been reported elsewhere [e.g. 47]. In particular, our results entirely agree with those found by Moreira et al. [48], who observed that simulating herbivory by MJ application increased the phosphorus and nitrogen concentrations in the shoots while maintaining or reducing those in the roots of *P. pinaster* juveniles, suggesting an active reallocation of nutrients to aboveground parts after herbivory signaling.

We found that the inducibility of resin in the phloem differed across plant parts, whereas this effect was not seen when considering the whole stem resin content, or with phenolics in the needles (no interactive effects). So, our results apparently provide little support to the ODT prediction that plant parts that have high tissue value and are most frequently attacked should have high constitutive levels of defense and low inducibility, and vice-versa [6], [10]. This ODT prediction is based fundamentally on the trade-off commonly found between constitutive and induced defenses [10], [49]; namely that when the investment in constitutive defenses of a plant increases, inducibility is expected to decrease, and vice-versa. However, it is noted that the studies that led to this prediction were conducted on herbaceous plants in grasslands [6], [10], that can strongly differ from long-lived woody plants in terms of their defensive strategies. In fact, other studies with woody species have also produced apparently contradictory results, perhaps because there is relatively little information available regarding inducibility of different chemical compounds in neighboring plant tissues. For instance, Tomlin et al. [50] found that following simulated weevil wounding, volatile terpenes and diterpene resin acids in the xylem of white spruce leaders markedly increased in the upper parts while decreasing in the lower region of the leader, but they found no significant changes in the concentration of those compounds in the upper and lower bark [50]. In other words, although they found that the pattern of resin allocation differed among susceptible and resistant lineages, they did not find the upper leader of spruce trees, the target tissue for weevils, having high constitutive levels and low inducibility, as the ODT would predict. It is likely that unrecognized genetic variation in defensive strategies of allocation among tissues, chemical and plant parts can explain the observed phenotypic variation, as recognized elsewhere [29], [46]. In addition, valuable plant parts may be also protected by other kinds of chemical defense (defensive proteins, volatile terpenes, alkaloids…) that were not measured in this study, and furthermore differential inducibility across plant parts could occur at the level of specific chemical compounds. Indeed studies about predictions of the ODT should probably consider a multivariate space of chemical defenses, including induced indirect defenses [51], [52] and nutrient induced resource reallocation, as well as other factors such as the indirect costs involved in the balance between costs and benefits, taking into account that genetic variation in most of those traits in increasingly recognized.

In summary, our results showed a marked pattern of quantitative chemical defense allocation along the pine juveniles (between upper apical and basal parts) and among different plant tissues (needles and stem). This fact could reflect differences in tissue value and risk of attack, although it could also reflect other non-adaptive causes such as ageing tissues or ontogenetic constraints. In accordance with ODT predictions, we found that upper stem tissues had the highest tissue value (highest levels of nitrogen) and thus were more defended with resin compounds than lower sections. Contrary to ODT predictions, however, we found that needle tissues had better developed chemical defenses (both resin and phenolic compounds) in the basal parts of the plant, despite their lower tissue value (low N concentration). We found greater inducibility of phloem resin in the lower part of the stem, and, even though some genetic variation in inducibility was found, the inducibility of other chemical defenses in response to jasmonate signaling did not differ among plant parts or tissues.
